# Antibacterial, Antifungal, Antiviral Activity, and Mechanisms of Action of Plant Polyphenols

**DOI:** 10.3390/microorganisms12122502

**Published:** 2024-12-04

**Authors:** Slavena Davidova, Angel S. Galabov, Galina Satchanska

**Affiliations:** 1 UPIZ “Educational and Research Laboratory”-MF, NBU, Department Natural Sciences, New Bulgarian University, Montevideo Blvd., 21, 1618 Sofia, Bulgaria; stdavidova@nbu.bg; 2 Stephan Angeloff Institute of Microbiology, Bulgarian Academy of Sciences, Acad. G. Bonchev Str., Bl. 26, 1113 Sofia, Bulgaria; galabov@microbio.bas.bg

**Keywords:** polyphenols, plants, bacteria, fungi, viruses

## Abstract

This review describes the enhanced classification of polyphenols into flavonoids, lignans, phenolic acids, stilbenes, and tannins. Its focus is the natural sources of polyphenols and an in-depth discussion of their antibacterial, antifungal, and antiviral activity. Besides a broad literature overview, this paper contains authors’ experimental data according to some daily consumed vegetables such as tomatoes, different varieties of onion, garlic, parsley, and cayenne pepper and the probable relation of these activities to polyphenols. The isolation of polyphenols via conventional and ultrasonic, pressurized liquids and pulse-field extractions, as well as their methods for detection and determination, are interpreted as well. The main mechanisms by which polyphenols inhibit the growth of bacteria, fungi, and viruses, such as protein synthesis, cell membrane destabilization, and ROS production induction, are in focus. Data on polyphenol concentrations and their respective MIC or the inhibition zone diameters of different bacterial and fungal species and suppressing viral replication are depicted. The toxicity of polyphenols in vitro, ex vivo, and in vivo towards microorganisms and human/animal cells, and the safety of the polyphenols applied in clinical and industrial applications are expanded. This review also characterizes the antimicrobial effects of some chemically synthesized polyphenol derivatives. Biotechnological advances are also reported, especially the entrapment of polyphenols in biocompatible nanoparticles to enhance their bioavailability and efficacy. Polyphenols are promising for exploring molecules’ novel antimicrobial substances and paving the path for effective novel antimicrobial agents’ discovery, taking into consideration their positives and negatives.

## 1. Introduction

The rise in antibiotic resistance over the past two decades has emerged as a significant global threat to humanity. The rise in multidrug resistance in pathogenic bacteria jeopardizes the effectiveness of antibiotics, which have historically revolutionized medical science. The challenge of antimicrobial resistance has been linked to the improper use of these drugs and the lack of new treatments due to stringent regulatory standards and diminished financial incentives. Extensive efforts are essential to slow the development of resistance by investigating new microorganisms, their resistance mechanisms, and various antimicrobial agents [1]. A multidisciplinary approach is necessary across healthcare systems, as well as in environmental and agricultural sectors. Innovative alternative strategies, including probiotics, antibodies, vaccines, and polyphenols, have demonstrated encouraging outcomes in trials, indicating their potential as preventive or supplemental therapies in the future. Recently, the worldwide use of antimicrobials in livestock has highlighted tje areas with high antibiotic consumption around the globe, which could have economic and public health repercussions in the future. In food-producing animals, antibiotics are typically utilized in cattle, chickens, and pigs, and it is anticipated that by 2030, this usage will rise by as much as 67% in the most densely populated nations [1].

Plants serve as a valuable source of new antimicrobials, and their secondary compounds, such as polyphenols, exhibit potent antimicrobial properties even at low concentrations [2]. Polyphenols are derived from phenol, which consists of an aromatic ring and a hydroxyl group.

These complex substances are polyaromatic and contain multiple hydroxyl groups. They can be categorized into five main groups: flavonoids, lignans, phenolic acids, stilbenes, and tannins, with flavonoids being the most abundant [3,4]. Polyphenols protect plants against bacteria, viruses, fungi, insects, and herbivores. Their synthesis originates from two aromatic amino acids—tyrosine and phenylalanine. As secondary metabolites in plants, polyphenols constitute only about 10% of plant metabolites [5,6].

**Figure 1:**
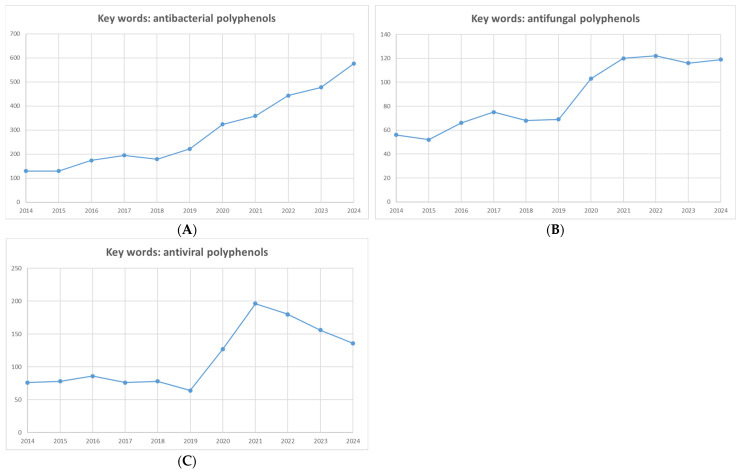
Scheme displaying three graphs showing the number of publications over ten years (2014–2024): (**A**) number of publications found using keywords “antibacterial polyphenols”; (**B**) number of publications found using keywords “antifungal polyphenols”; (**C**) number of publications found using keywords “antiviral polyphenols”. Search was conducted using PubMed (https://pubmed.ncbi.nlm.nih.gov/ accessed on 11 October 2024). During the search, it was observed that the amount of articles published on the topic of antiviral activity for polyphenols in 2020 was 64, whereas, in the next two years, during the peak of the COVID-19 pandemic, the published articles were 153 and 127, respectively.

Plants have always provided valuable bioactive substances that are used worldwide to treat various diseases. Identifying and analyzing the compounds found in herbs has played a role in uncovering new pharmaceutical resources [7]. Approximately 11% of the essential drugs available globally are derived solely from plants. Unlike their synthetic counterparts, natural preparations exhibit anti-inflammatory, antioxidant, and anticancer properties in various ways, making them safer for patients as they have no side effects [8,9]. Polyphenols are secondary metabolites ubiquitously distributed in all higher plants, which play an essential role in defense against plant pathogens and animal herbivore aggression and as a response to various abiotic stress conditions, such as rainfall and ultraviolet radiation [10]. The use of polyphenols as antimicrobials has been widely discussed, and interest in the topic has increased significantly in the last ten years. Figure 1 shows the increase in publications on using antibacterial, antifungal, and antiviral polyphenols as novel antimicrobials.

**Figure 2:**
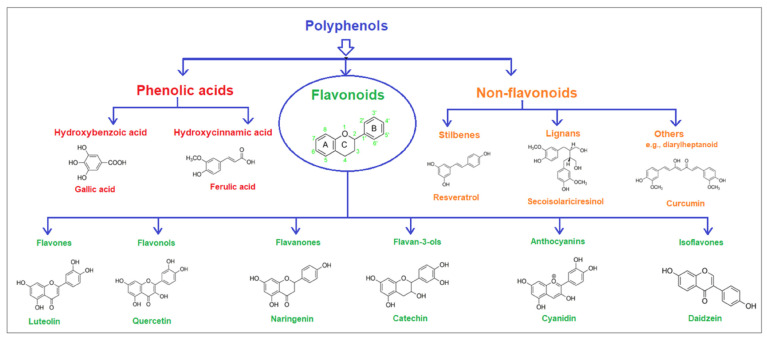
Classification of polyphenols [12]. Different colors represent the different classes of polyphenols.

Polyphenols are natural compounds with multiple hydroxyl groups and aromatic rings. They are primarily found in fruits, vegetables, cereals, and beverages [11]. They are generally classified into three main groups: phenolic acids, flavonoids, and non-flavonoids (Figure 2) [12]. The polyphenol content in fruits (red raspberry, blackberry, strawberry, and apple) ranges from 1.5 to 3 mg/g of fresh weight. The antimicrobial activity of fruit extracts such as *Pyrus communis*, *Malus pumila*, *Prunus domestica*, *Prunus persica*, *P. avium*, *Prunus cerasus vulgaris*, *Rubus idaeus*, *Fragraria vesca*, *Vinis vinifera*, *Cornus mas*, *Rubus fruticosus*, *Viccinum mirtilus*, and *Ficus carica* has been tested. The highest polyphenol content is in *Viccinum mirtilus* (European black berry)—6.70 mg GAE/g of fresh mass followed by *C. mas* (dogwood)—at 4.29 mg/g, and the lowest content was demonstrated *by P. persica* (peach) at 0.50 mg GAE/g [5].

Vegetables that are rich in bioactive compounds include black or green olives (5.69 mg/g and 3.46 mg/g, respectively), artichokes (2.6 mg/g), and chicory (2.35 mg/g). Tea (black, 102 mg/100 mL; green, 89 mg/100 mL), red wine (101 mg/100 mL), and rapeseed oil (92 mg/100 mL) can also be sources of polyphenols [8]. According to their chemical structure, dietary polyphenols are classified into five major classes, including phenolic acid (hydroxybenzoic acid, hydroxycinnamic acid), stilbenes, lignans, flavonoids (flavanols, isoflavones, anthocyanins, flavanones, flavones, flavonols), and tannins [13].

The flavonoids in fruits, vegetables, herbs, spices, and medicinal plants are widespread polyphenols responsible for the vibrant pigments in these natural products [14] and they are recognized as dietary supplements that support health and help prevent illnesses [13]. They consist of two aromatic rings connected by a three-carbon bridge with 15 carbons. Based on their C-ring structure differences, they are classified into various subgroups, including flavones, flavanones, isoflavones, flavonols, flavan-3-ols, and anthocyanidins. Each subgroup exhibits distinct characteristics due to variations in their hydroxylation, prenylation, glycosylation, or methoxylation patterns. Flavonoids include quercetin, catechin, naringenin, cyanidin–glycoside, and daidzein. They are present in different forms, such as free aglycones, glycosidic conjugates, and modified forms [4].

The combination of two C6-C3 units connected between positions 8 and 8′ is known as lignan, a non-flavonoid compound. Lignan C9 and C9′ are substituted in different patterns, resulting in various structural forms. Lignans can be categorized into subgroups such as furan, dibenzylbutane, and aryltetralin [15]. Lignans are predominantly found in free form in legumes, seeds, and vegetable oils, as glycosylated structures are uncommon. Lignin is an aromatic biopolymer formed through the phenolic oxidative coupling of p-hydroxycinnamoyl alcohol monomers by peroxidase enzymes. The primary alcohols involved are 4-hydroxycinnamoyl, coniferyl, and sinapoyl [4]. An increasing number of studies on lignans have highlighted their diverse pharmacological effects, particularly concerning their role in cancer chemotherapy [13].

Phenolics are a type of non-flavonoid polyphenol that have multiple OH groups on aromatic rings. Phenolic acids have two main structures: benzoic acid, which contains seven carbon atoms, and cinnamic acid, which contains nine carbon atoms [16]. Hydroxybenzoic acids, a rare component of the human diet, are not believed to impact human health significantly. The derivatives of benzoic acid include p-hydroxybenzoic acid, protocatechuic acid, salicylic acid, gallic acid, and ellagic acid. The derivatives of cinnamic acid include p-coumaric acid, caffeic acid, and ferulic acid. Phenolic acids are essential components of the human diet and have various health benefits, such as anti-microbial, antioxidative, anti-inflammatory, immunoregulatory, anti-allergic, anti-atherogenic, cardioprotective, anticancer, and antidiabetic properties [4].

Stilbenes, another class distinct from flavonoids, consist of two phenyl groups connected by a 2-C methylene bridge. They are composed of two aromatic rings, A and B, and are found in various forms, including isomeric (*cis*- and *trans*-), free, and glycosylated forms [4]. Ring A of stilbenes contains two hydroxyl groups at the m-position, while ring B has several positions substituted with hydroxy and methoxy groups. Resveratrol (3,5,4′-trihydroxystilbene) is a well-known stilbene compound produced by berries, grapes, and nuts [16]. As the key compound among stilbenes, the resveratrol found in red wine has been proposed to help prevent chronic illnesses and, therefore, provide health advantages [13,17].

Tannins are compounds with high molecular weights that range from 500 Da to 30,000 Da, and they can be categorized into two primary types: condensed and hydrolysable tannins. Condensed tannins, which include proanthocyanidins (1−30 kDa), are composed of oligomers and polymers made up of flavan-3-ol and flavan-4-ol subunits, with catechin, epicatechin, and leucoanthocyanidins serving as their main components [13]. Procyanidins can be subdivided into A-type, characterized by double linkages (C-C and C-O), and B-type, which are distinguished by interflavanol linkages. These compounds resist acid hydrolysis and can be digested in the gastrointestinal tract thanks to the stable C-C linkages. Hydrolysable tannins (500−3000 Da) are polyester compounds from a combination of glucose with gallic or ellagic acid [13]. Acids, bases, and enzymes can hydrolyze them and are further divided into gallotannins and ellagitannins. Gallotannins consist of polygallic glucose esters, whereas ellagitannins are connected through hexahydroxydiphenoyl (HHDP) units. Tannins can be found in various plants and foods, including cereals, fruits, and beverages, where they play a role in plant defense mechanisms and may offer potential health benefits [13].

**Figure 3:**
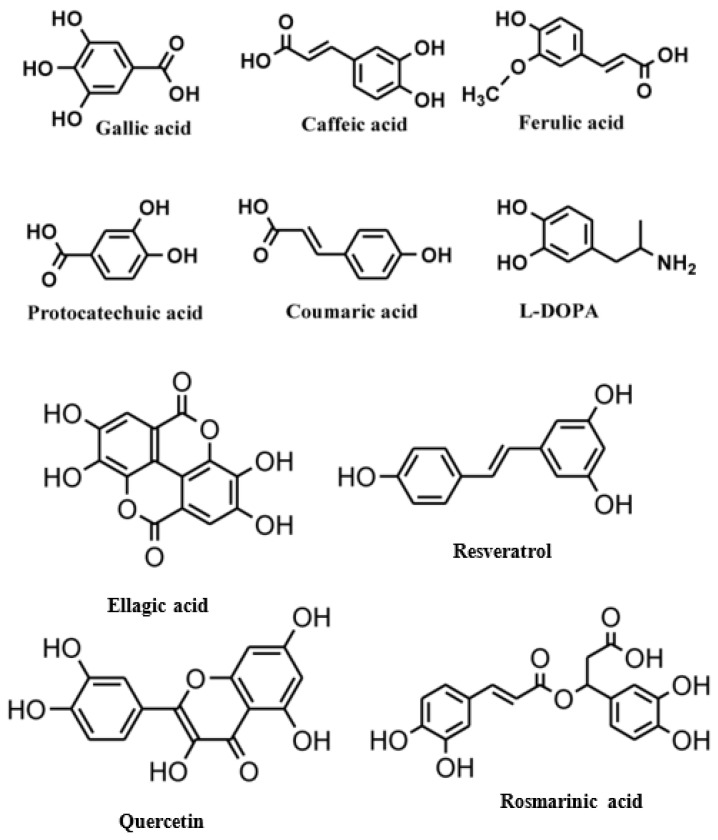
Structural formulas of the most common plant polyphenols with pronounced antibacterial, antifungal, and antiviral activity [20].

The pharmaceutical, nutraceutical, cosmetic, and food industries have shown significant interest in plant polyphenols because of their diverse biological activities. These plant-derived secondary metabolites, abundant in plants, not only represent a promising area of research, but also show an immense potential for practical applications. They are further divided into phenolic compounds, phenolic acids (cinnamic acid derivatives), and glycosidic phenylpropanoid esters. Previous studies have shown that phenolic compounds possess antimicrobial and anti-inflammatory properties, and their antioxidant effects [18], attributed to the hydroxyls attached to aromatic rings, make them effective scavengers of reactive oxygen species and the chelators of Fe^3+^ that catalyze lipid peroxidation. The potential of plant polyphenols presents exciting possibilities for future applications [19]. Figure 3 below shows the structures of common plant polyphenols.

## 2. Antibacterial Polyphenols

Polyphenols exhibit antibacterial properties against various bacteria, encompassing Gram-positive and Gram-negative bacteria [21]. A significant category of bacterial strains is resistant to multiple drugs, creating a specific hazard for individuals employed in nursing facilities and hospitals [22]. Many microorganisms (for instance, *Staphylococcus aureus*, *Escherichia coli*, *Pseudomonas aeruginosa*, *Acinetobacter* sp., *Proteus* sp., *Micrococcus* sp., *Klebsiella pneumonia*, *Staphylococcus epidermidis*, *Bacillus subtilis*) endanger our lives daily. They are susceptible to polyphenolic compounds (phenolic acids, flavonoids, and non-flavonoids) such as catechin, phenolic acid, cinnamic acid, ferulic acid, sinapic acid, p-coumaric acid, epigallocatechin gallate, daidzein, resveratrol, and curcumin, etc. [12]. *Curcuma longa* L. is a perennial plant from the *Zingiberaceae* family, commonly called turmeric. Curcumin restrains the growth of numerous bacteria, including *Staphylococcus aureus*, *Salmonella paratyphi*, *Bacillus subtilis*, *B. macerans*, *B. licheniformis*, and *Azotobacter* [23]. The antimicrobial properties of curcumin nanoparticles were evaluated against *S. aureus* (ATCC 333591) and *P. aeruginosa* (MTCC 4676) using the Kirby–Bauer disk diffusion method. Curcumin nanoparticles were observed to be most effective against *P. aeruginosa* (12 mm) and less effective against *S. aureus* (10 mm) [23].

Salicylic acid inhibits the growth of different fungi and bacteria, including *Propionibacterium acnes*, associated with acne formation [24]. It disturbs bacterial membranes and obstructs bacteria’s metabolism. In Adamczak et al.’s work, salicylic acid inhibited bacterial growth with the MIC being between 250 and 500 μg/mL [25].

Gallic acid, ellagic acid, and 4-hydroxybenzoic acid eliminate microbial agents by disrupting cell walls and metabolic processes, thus impeding enzyme activity and effectively combating bacteria and fungi [12,26].

P-coumaric acid is the primary antibacterial component of alkaline hydrolysates compared to caffeic and ferulic acid. Additionally, hydroxycinnamic acids also display antibacterial effects [27]. Notably, p-coumaric acid, with IC50 levels ranging from 200 to 400 µg/mL, exhibits a more potent impact against various enterobacterial strains.

Apigenin demonstrates considerable antibacterial and antibiotic–synergistic action against oral infections. Previous studies have shown the significant inhibition of bacterial growth by luteolin, baicalein, and tangetin. Luteolin is a more effective antimicrobial agent through the inhibition of nucleic acid and protein synthesis, the impairment of bacterial cell membranes, the modification of cell morphology, and the inhibition of bacterial biofilm formation. Catechin and arbutin limit the growth of *Salmonella typhimurium* ATCC 13311. Phloretin and phlioridzin not only kill microorganisms, but they also reduce lymphatic node metastases. Anthocyanins inhibit the growth of *Listeria monocytogenes*, *Staphylococcus aureus*, *Bacillus subtilis*, *Enterococcus faecalis*, and yeast strains (*Debaryomyces hansenii*, *Trichosporon cutaneum*, etc.). The strains susceptible to resveratrol (50 µg/mL) and curcumin (20 µg/mL) at sub-MIC levels show at least a 50% reduction in virulence factors (IC50) in antimicrobial agent experiments. In most isolations (57–94%), the hemagglutination and hemolysin activities were reduced by 50%. Protease and biofilm IC50 values were observed in 6.5–17.8% of the isolates using a methicillin-resistant strain of *Staphylococcus aureus* [12].

Baicalein has been found to hinder replication with cell adhesion in *S. aureus*. In a study by Chen et al., it was shown through plate counting, crystal violet staining, and fluorescence microscopy that baicalein concentrations of 32 μg/mL and 64 μg/mL significantly hindered the formation of *S. aureus* biofilms after 3 and 7 days in vitro [28].

Catechin epigallocatechin-3-gallate’s (EGCG) ability to bind to porins is likely the reason for its influence on damaging the outer cell membrane of Gram-negative bacteria through porin pores. Flavonoids have successfully killed bacteria like *Escherichia coli* by creating extracellular and dissolved protein complexes [12]. Table 1 depicts the various foods we consume daily that are rich in polyphenols. Although those foods are rich in polyphenols, dieting with only those foods is insufficient for antibacterial, antiviral, and antifungal effects.

The health benefits of olives result from their richness in the polyphenols commonly found in Mediterranean fruits and vegetables, which are part of the Mediterranean diet. The primary phenolic compound responsible for their positive effects on health is hydroxytyrosol [29,32]. In addition to its antimicrobial properties, hydroxytyrosol is a potent antioxidant and radical scavenger, leading to apoptosis and cell cycle arrest in cancer cells. Usually, the body eliminates hydroxytyrosol through the kidneys. Other phenolic compounds in olives include tyrosol, glycoside oleuropein, oleocanthal, and oleacein. Both hydroxytyrosol and oleuropein have been shown to exhibit antimicrobial activity against ATTC bacterial strains and clinical bacterial isolates [29,33].

Rosemary, scientifically known as *Rosmarinus officinalis* L., is a fragrant herbaceous plant from the *Lamiaceae* family. It is indigenous to the Mediterranean region but is grown globally. Rosemary is extensively utilized in traditional medicine as a spice, preservative, and herbal medicine. Due to its high polyphenol content, rosemary is an antioxidant and preservative in cosmetics, food products, and other complex systems [34]. Additionally, it is used as a natural remedy for safeguarding against various degenerative diseases linked to oxidative stress, such as cancer, heart disease, neurodegenerative disorders, diabetes, age-related skin damage, and osteoporosis. The biological properties of rosemary are attributed to the presence of the phenolic compounds from three primary classes: phenolic diterpenes (e.g., carnosic acid, carnosol, rosmanol, epirosmanol, and methyl carnosate), flavonoids (e.g., cirsimaritin, genkwanin), and phenolic acids (e.g., rosmarinic and caffeic acids), among others, highlighting the herb’s versatility and potential across various applications [34,35]. Mihajilov-Kristev et al. [36] demonstrated that essential oils rich in carvacrol (67.0%) and γ-terpinene (15.3%) were effective against Gram-negative strains, inc luding *Escherichia coli*, with the MIC values ranging from 0.025 µL/mL to 0.78 µL/mL as determined by the broth microdilution method. Probuseenivasan et al. [37] verified that rosemary essential oil strongly inhibits *E. coli* ATCC 25922. The minimal inhibitory concentration for rosemary oil against *E. coli* was >6.4 mg/L [38].

Sea buckthorn (SBT) (*Hippophae rhamnoides*) is an economically significant prickly shrub with separate male and female plants. Different parts of male and female SBT plants contain compounds with diverse biological properties. The highest phenolic content is found in the leaves, which display antioxidant and antibacterial effects. SBT leaves are abundant in phenolics, flavonoids, tannins, carotenoids, tocopherols, free and esterified sterols, and triterpenols, offering a range of health benefits such as antioxidant, cytoprotective, antibacterial, antitumor, antiviral, anticancer, immunomodulatory, anti-inflammatory, radioprotective, adaptogenic, and anti-cardiovascular properties [39]. Phenolic compounds are recognized as the primary contributors to the biological activity of SBT leaves. Despite the presence of valuable bioactive compounds, most SBT leaves are discarded as agricultural waste following berry harvesting. These leaves are rarely utilized in food production due to its lack of widespread recognition as a food or food supplement. Efforts have been made to introduce the scope and procedure of Novel Food Regulations to utilize sea buckthorn leaves as food, food supplements, or spices. Thus, it is crucial to extract, measure, and identify the bioactive compounds from the unused leaves and promote their subsequent application based on their antimicrobial, antioxidant, and anticancer properties [39].

Honey is commonly known as a highly concentrated sugar solution primarily composed of fructose and glucose. However, it is also a complex combination containing proteins from plants and bees and polyphenols, mainly flavonoids, antioxidants, and Maillard reaction products [40,41]. Extensive research has characterized honey’s wide-ranging antimicrobial properties against fungi and bacteria [42]. Due to the production of hydrogen peroxide (H_2_O_2_) from bee-derived glucose oxidase, which becomes activated when diluted with water, many types of honey display strong antimicrobial properties [40].

Seaweeds have the potential to be valuable sources of bioactive compounds and phytochemicals for use as supplements in animal feed, and they are rich in polyphenols [43,44]. The brown seaweed *Ascophyllum nodosum* demonstrated significant antioxidant and antibacterial properties. The results of a study by Hejna et al. suggest that the bioactive molecules from *Ascophyllum nodosum* exhibit potent inhibitory effects on *E. coli* strains. Further investigation is necessary to investigate the therapeutic potential and possibilities of the compounds derived from algae [43].

The consumption of tomatoes is the second highest among all the vegetables worldwide. The author’s previous results indicated no significant variance in the impact of raw or cooked tomato products on bacteria [29]. The antibacterial effect was relatively weak, with a maximum zone of 7 mm. Among the six tomato varieties, the seeds of two varieties marginally restricted the growth of *B. subtilis* and did not exhibit any antibacterial activity against *E. coli*. On agar plates, the inhibition zones measured between 4 and 7 mm [45]. Tomatoes are rich in polyphenols such as naringine chalcone (0.9–18.2), rutin (0.5–4.5), quercetin (0.7–4.4), chlorogenic acid (1.4–3.3), naringenin (0–1.3), kaempferol-3 rutinoside (0–0.8), p-coumaric acid (0.2–0.5), ferulic acid (0.2–0.5), and kaempferol (0–0.2) [29,46].

*Allium sativa* (onion) has strong antibacterial effects against Gram (−) *E. coli* (30 mm) and weaker effects against Gram (+) *B. subtilis* (7 mm). *Allium cepa* (garlic), rich in polyphenols, has lower antibacterial activity than garlic against the strains tested. *A. cepa* contains anthocyanins and flavonols, two types of flavonoids [45]. Anthocyanins are responsible for the red color of certain onion varieties, while flavonols such as quercetin contribute to the orange-brown color of onion skin. Over 25 different flavonols are found in onions, with quercetin in all the varieties. Quercetin 4′-glucoside and quercetin 3,4′-glucoside account for more than 80% of the total flavonols in *A. cepa* [29].

The phenol content of cayenne pepper is impressive [47]. Our tests on cayenne pepper fruits and seeds demonstrated that they inhibit the growth of *E. coli* and *B. subtilis*. When the pepper tissues were crushed, no antibacterial effect was observed. However, the pepper disks showed significant antibacterial activity against *E. coli* (25 mm) and *B. subtilis* (24 mm). It was interesting to note that, similar to tomato seeds, pepper seeds also inhibited the growth of *E. coli* (11 mm) and *B. subtilis* (7 mm) [29,45].

Parsley, a plant utilized as a medicinal plant, spice, and herb, has displayed antibacterial properties. It contains various polyphenols, primarily flavonoids, such as kaempferol, apigenin, and luteolin [48]. The average flavonoid content is around 100 mg per 100 g of fresh weight. Crushed green parsley leaves exhibited a mild antibacterial effect against *E. coli* and *B. subtilis* [29]. Table 2 presents the original results, testing the antibacterial activity of parsley, tomato, cayenne pepper, onion, and garlic.

The antibacterial activity of different plants against Gram-positive *B. subtilis* and Gram-negative *E. coli*, as shown in Table 2, could be due to the presence of polyphenols or antimicrobial peptides or their synergetic activity in plants, demonstrating substantial activities against various pathogens. The highest antimicrobial action on *B. subtilis* was shown by yellow skin onion (27 mm), red skin onion (25 mm), and cayenne pepper (24 mm), and the highest antimicrobial action on *E. coli* was demonstrated by garlic (30 mm) and cayenne pepper (25 mm) [48].

Polyphenols positively impact the gastrointestinal tract due to their ability to either inhibit the growth of specific pathogens or kill them. They can hinder the growth of various pathogenic bacteria such as *Helicobacter pylori*, *Pseudomonas aeruginosa*, *Escherichia coli*, *Streptococcus mutans*, *S. aureus*, *Salmonella enteritidis*, *Vibrio cholerae*, *Klebsiella pneumoniae*, *Yersinia enterocolitica*, *Listeria monocytogenes*, *Bacteroides fragilis*, *Clostridium perfringens*, and *Clostridium difficile.* Additionally, they can be used as food additives to prevent the multiplication of antibiotic-resistant bacteria colonizing in foods [49]. Our previous experiment showed that *Enterococcus faecalis*, found in cheese, was resistant to three antibiotics belonging to the class Fluoroquinolones (Ciprofloxacin, Levofloxacin, and Norfloxacin) [50,51]. Another great advantage of polyphenols is that some of them can promote the growth of beneficial bacteria. For instance, the polyphenols from grapes can enhance the growth of *Akkermansia muciniphila*. In contrast, rutin, resveratrol, cocoa polyphenols, blueberry anthocyanidins, apple procyanidin B2, and chlorogenic acid can stimulate the growth of *Lactobacillus* and *Bifidobacterium* [52].

## 3. Antifungal Polyphenols

The potent antifungal properties of polyphenols are supported by substantial evidence [53]. As per Manso et al., the extracts from *Acacia nilotica* (babul), *Cinnamomum zeylanicum* (cinnamon), and *Syzygium aromaticum* (clove) demonstrated antifungal effects against *C. albicans* [21]. The extracts from *A. nilotica* exhibit MIC values in the range of 0.158 mg/mL, having a zone of inhibition of 37.5 ± 0.143 mm [54]. The extract from the leaf of *C. zeylanicum* had a MIC of 0.63 μg μL^−1^ against *C. albicans* [55]. *S. aromaticum* showed an inhibition zone diameter of 29.62 against the oral isolates of *C. albicans* from patients [56].

The extracts derived from the leaves and inflorescences of various species of *Amaranthus retroflexus* (red-root amaranth) were also effective against fungi *Candida famata*, *Candida utilis*, *C. albicans*, and yeast *Saccharomyces cerevisiae*, particularly impacting *C. famata* [21]. It has also been reported that gallic acid, ellagic acid, and corilagin (Table 3) show activity against *Candida* species and *Cryptococcus neoformans*. The MIC of gallic acid against C. glabrata, *C. albicans*, and *C. troplicalis* was reported to be between 12 and 100 μg/mL [57]. Ellagic acid and the extracts from longan (*Dimocarpus longan Lour*, Dragons’ eye) seeds were effective against *C. albicans* and *C. neoformans*. Corilagin and ellagic acid displayed mild antifungal activity against *Trichophyton rubrum*, *Microsporum gypseum*, and *Epidermophyton floccosum*, while longan seed extract had a slight antifungal impact on the tested dermatophyte species [21].

The red seaweed *Jania rubens* has been found to inhibit the growth of various bacteria, fungi, and viruses. Additionally, quercetin, rutin, morin, and myricetin prevent fungal cell growth [58]. Galangin and kaempferol, commonly found in propolis samples, exhibit inhibitory effects against *Aspergillus tamarii*, *Aspergillus flavus*, *Cladosporium sphaerospermum*, *Penicillium digitatum*, and *Penicillium italicum*. Treatments with naringenin and naringin reduce lipid peroxidation and increase antioxidant levels in rats. Hesperidin and eriodictyol demonstrate pharmacological effects, including broad antifungal properties, with the highest antifungal activity observed for eriodictyol. As reported by Mandal et al., daidzein and genistein affect *C. albicans* and *T. rubrum*, while glycitein possesses effective antifungal properties [12]. Exceptionally effective is curcumin, which has shown unique antifungal activity against 20 *Candida* species [23].

Recent research has also highlighted the antifungal effects of flavonols. It has been demonstrated that propolis, a globally recommended external topical treatment for relieving various fungal skin disruptions caused by fungal strains, was active against *Microsporum gypseum*, *Trichophyton mentagrophytes*, and *Trichophyton rubrum*. The primary active components responsible for this antifungal activity were flavonols such as galangin, izalpinin, and rhamnocitrin. In addition to flavonols, other active propolis polyphenols included flavanones (pinocembrin and pinostrobin) and chalcones (2,4-dihydroxychalcone and 2,4-dihydroxy-3-methoxychalcone), which other studies have reported to possess antimicrobial properties with MICs between 16 and 125 μg mL^−1^ [10]. A recent study revealed that the ellagitannins derived from *Ocotea odorifera*, a medicinal plant commonly used in Brazil, demonstrated significant activity against *Candida parapsilosis* at a concentration of 1.6 μM.

The grapevine (*Vitis vinifera* L.) is one of the most widely grown fruit crops globally [59]. The ethanolic extracts from *V. vinifera* L. tendrils exhibit in vitro antifungal activity against plant pathogenic fungi, such as *Fusarium* species, with minimum inhibitory concentration (MIC) values ranging from 250 to 300 ppm. The basidiomycete fungus *Rhizoctonia solani* is the most resistant, with a MIC value of 500 ppm. Numerous studies have been published on the antifungal activity of *V. vinifera* extracts from grape wastes and the byproducts derived from agricultural and agro-industrial processes against human pathogens, most focusing on *Candida* [60].

According to Elansary et al., the stem extracts of *Ferocactus* sp. demonstrate strong antifungal properties against fungi. The reported MIC and minimum fungicidal concentration (MFC) values were consistently low for all the species of *Ferocactus* and were between 0.12 and 1.31 mg mL^−1^. The extracts showed outstanding antifungal effects against *Aspergillus ochraceus* and *A. niger*. However, *Penicillium funiculosum*, *P. ochrochloron*, and *Candida albicans* exhibited higher resistance. It should be highlighted that the effectiveness of the extracts was similar to that of the commercial antifungal agent ketoconazole (KTZ). The antifungal activities of the phenolic standards 3,4-dihydroxyphenylacetic acid, rutoside, and quercitrin were comparable to those of the extracts from *F. glaucescens*, *F. emoryi*, and *F. pottsii* [61].

**Table 3:** Structural formulas of other plant polyphenols with emphasized antibacterial, antifungal, and antiviral activity.

Polyphenol	Structure	Active Against	Reference
Salicylic acid	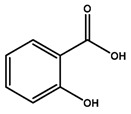	*Propionibacterium acnes*	[62]
Cinnamic acid	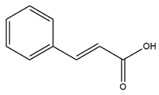	*S. aureus*, *E. coli*, *P. aeruginosa*, *Acinetobacter* sp., *Proteus* sp., *Micrococcus* sp., *K. pneumonia*, *S. epidermidis*, *B. subtilis*	[63]
Sinapic acid	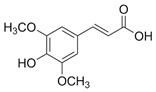	*S. aureus*, *E. coli*, *P. aeruginosa*, *Acinetobacter* sp., *Proteus* sp., *Micrococcus* sp., *K. pneumonia*, *S. epidermidis*, *B. subtilis*	[64]
EGCG	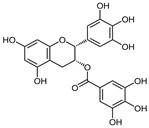	HCV, HIV, FLU, DENV, Chikungunya virus, ZIKV, SARS-CoV-2	[65]
Apigenin	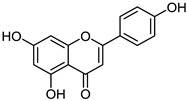	*B. subtilis*	[66]
Baicalein	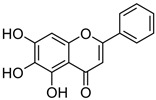	*S. aureus*	[67]
Phloretin	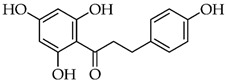	*E. coli*, *S. aureus*	[68]
Carnosic acid	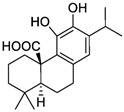	*E. coli*	[69]
Carnosol	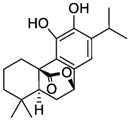	*E. coli*	[69]
Rosmanol	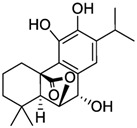	*E. coli*	[69]
Epirosmanol	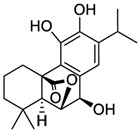	*E. coli*	[69]
Corilagin	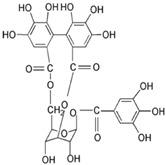	*Candida* sp., *Cryptococcus neoformans*, *Trichophyton rubrum*, *Microsporum gypseum*, and *Epidermophyton floccosum*	[70]
Myricetin	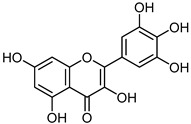	SARS-CoV-2, FLU	[71]

## 4. Antiviral Polyphenols

Viruses can quickly devastate our lives. The most prevalent viral pathogens include the flu, rhinovirus, adenovirus, coronavirus, respiratory syncytial (RSV), and SARS-2 viruses [72]. These viruses are responsible for various illnesses. Polyphenols such as resveratrol, luteolin, ellagic acid, and cyanidin can help destroy harmful viruses like hepatitis B and influenza, potentially saving lives [73]. These vital polyphenolic compounds can also offer protection against microorganisms like bacteria, fungi, and viruses [12].

Natural compounds benefit from being evolutionarily pre-selected with optimized chemical structures to target biological processes. One such compound is EGCG, a polyphenol found in green tea that makes up 59% of all polyphenols and has demonstrated various health benefits, including antiviral, anti-tumor, and antioxidant properties. EGCG has been shown to have antiviral effects against several viruses, such as the hepatitis C virus (HCV), the human immunodeficiency virus (HIV), the influenza virus (FLU), the Dengue virus (DENV), and the rare chikungunya virus [74]. As shown by in silico experiments, a recent study demonstrated that EGCG can destroy the Zika virus (ZIKV), possibly by inhibiting its entry into host cells. Kumar et al. also reported that EGCG may impede crucial stages in the replication cycle of certain viruses, like ZIKV, as they found that using an enzymatic assay has shown the significant inhibition of NTPase activity with an IC_50_ value of 295.7 nM and a Ki of 0.387 ± 0.034 μM [74].

The extracts of black willow (*Salix nigra*) were found to contain various polyphenolic compounds through phytochemical analysis [75]. They demonstrated antiviral activity against HCV in stable Hep G2 cell lines expressing the HCV NS3 gene. The results indicated that the extracts of *S. nigra* notably decreased the expression of the NS3 gene at both the mRNA and protein levels. The results of the cytotoxicity tests indicated that the methanol and water extracts of *S. nigra* exhibited significant cytotoxicity, with CC_50_ values between 25 and 30 μg/mL. Following 24 h of treatment, it was observed that as much as 95% of the cells treated with the extract had died [75]. These findings contribute to the global fight against HCV by providing plant-based treatment options for an HCV cure. Additionally, curcumin, a polyphenol derived from turmeric, has been proven to hinder HCV entry by preventing viral attachment to host cells [75].

Berries contain a variety of bioactive compounds with antiviral properties. Black elderberry, also known as *Sambucus nigra*, is a component of Sambucol preparation and has demonstrated effectiveness against influenza. In addition to inhibiting virus adsorption and replication, blackcurrant extracts reduce the risk of complications and inhibit other harmful microorganisms. Blueberries and cranberries exhibit powerful antiviral effects due to their polyphenol content [8]. *Aronia melanocarpa* ethanol extract contains antiviral polyphenols like ellagic acid and flavonoids like myricetin, kaempferol, and quercetin. The compounds derived from chokeberry have shown significant effectiveness against the seasonal influenza viruses of the H1 and H3 type, with only 0.0625 mg inhibiting nearly 70% of the viruses. Aronia extract likely inhibits virus surface proteins, particularly HA [8].

The recent COVID-19 pandemic caused by SARS-CoV-2 affected more than six million people and caused thousands of deaths. Certain polyphenols, such as luteolin, strongly attract the coronavirus’s S protein, preventing the virus from entering human cells [76]. The inhibition of the S protein is primarily due to the polyphenols found in citrus fruits, turmeric, and rhubarb roots. Other scientific sources have suggested that the active compounds found in herbs and tea, such as naringenin, EGCG, and herbacetin, may also have the potential to block the S protein [77]. Furthermore, the IC_50_ of flavonoids such as luteolin, hesperetin, and quercetin ranges from 47 to 73 μM, which can obstruct critical proteins (PLpro, 3CLpro) that play a role in the SARS coronavirus infection cycle. Additionally, the viral ACE2 enzyme, which acts as the gateway for SARS-CoV-2, can be blocked to prevent entry into host cells [77]. The polyphenols in turmeric, yerba mate (South American, caffeine-containing), and red grapes (eriodictyol, resveratrol, curcumin, and catechin) have a strong affinity for ACE2 ligands [8]. Once the virus has entered the human body, the polyphenols halt its ssRNA replication. Its main focus is on inhibiting protease, which effectively blocks the transcription and replication of genetic material. The significance of the polyphenols from turmeric and citrus fruits has also been acknowledged. Conversely, polyphenols such as EGCG, myricetin, and quercetagetin strongly attract SARS-CoV-2 RdRp, an RNA polymerase that produces an RNA strand on the matrix [8]. Numerous herbs and natural products have demonstrated the inhibition of CoVs, but their antiviral mechanisms remain incompletely understood [78].

Black tea polyphenols have been shown to aid in the absorption of minerals in the intestine and exhibit antiviral properties. The theaflavins in black tea have been discovered to have anti-HIV-1 effects by preventing the entry of HIV-1 cells into host cells. The entry of HIV-1 into host cells involves the fusion of membrane glycoprotein (GP) and the virus’s envelope with the host cell membrane [79]. The heptad repeat units, located at the N and C terminals of GP41, merge to form the fusion-active GP41 core, a six-helical bundle. The heptad (HPPHCPC) consists of seven amino acids with hydrophobic (H), charged (C), and polar amino (P) residues [79]. It was found that theaflavins can prevent the formation of this six-helix bundle, which is necessary for the virus to enter the host. The IC_50_ of epitheaflavin-3′-gallate (TF_2_B) for inhibition HIV-1 replication is 1.04 ± 0.21 μM [79]. Additionally, theaflavin-3, 3′-digallate, and theaflavin-3′-gallate were found to inhibit the severe acute respiratory syndrome (SARS) coronavirus. This antiviral activity is attributed to the inhibition of the chymotrypsin-like protease (3CL Pro), which is involved in the proteolytic processing during viral replication [78,80].

## 5. Isolation of Polyphenols

The isolation of polyphenols presents a difficult challenge due to these compounds’ unstable and complex nature [81]. Polyphenols are primarily found in the plant leaves and gymnosperms within the cell wall and vacuoles and are associated with the nuclei. The covalent attachment of polyphenols to plant structures restricts their release [82]. In addition, various other factors impact the extraction of phenolic compounds from plant samples, such as their location in plant tissues, the extraction method used, the sample size, the storage conditions, and the possible subsequent chemical transformations. Water or organic solvents can obtain various plant secondary metabolites, including polyphenols [29].

### 5.1. Conventional Methods

Since polyphenolic chemicals are typically found in trace amounts in various foods, drinks, and plant matrices, extraction techniques are required. Pretreatment methods including crushing, grinding, and drying might be needed, depending on the samples. To acquire the active chemicals, extraction is followed by isolation and purification. Percolation, decoction, heat reflux extraction, Soxhlet extraction, and maceration are examples of conventional techniques. These differ based on the characteristics and makeup of the food samples. Despite their ease of use, conventional procedures have drawbacks such as lengthy extraction times, significant energy usage, and solvent waste [82].

The conventional extraction methods involve using solvents such as water, hexane, ether, chloroform, acetone, benzene, ethanol, and methanol to extract bioactive compounds from plant material. Ethanol dissolves alkaloids, glycosides, and dyes, but not gum, waxes, or fats. Acetone is effective for tannin extraction and prevents microbial growth. Chloroform, ether, and bischloromethanol are suitable for extracting specific compounds but have disadvantages such as volatility, flammability, explosiveness, and toxicity. The percentage of different solvents strongly influences the polyphenol yield. Guava is the most abundant in polyphenols [83]. The main steps of polyphenol extraction are sample grinding, extraction, filtration, concentration, and drying. Soxhlet extraction is preferable due to its high extract yield, low cost, and ease of conducting the process [29].

### 5.2. Non-Conventional Technologies

Several pieces of research have demonstrated the potential of traditional extraction procedures such as Soxhlet extraction and the maceration process, with promising results. However, such approaches need a lot of solvent, time, and energy. Ultrasound-assisted extraction, microwave-assisted extraction, and supercritical fluid extraction are three commercially available extraction procedures. These approaches have shown a promising potential for increasing polyphenolic content by 32–36% while using approximately 17.6 times less energy than heat treatments. Emerging approaches such as high-voltage electric discharge, pulse electric field extraction, and enzyme-assisted extraction have also gained popularity and have been studied in a variety of food and plant systems. These procedures demonstrate the high-quality extract with the least raw material and energy consumption [82].

Polyphenols are extracted using ultrasonic, microwave, pressurized liquid, and pulse electric field extraction methods [84,85]. HPLC-DAD (high-performance liquid chromatography with diode array detection) is the best method to determine polyphenols. This method has been used to analyze many phenolic compounds, such as epicatechin, vanillic acid, quercetin, kaempferol, epigallocatechin, rutin, and myricetin. The structure elucidation of polyphenols can be implemented using gas chromatography-GM spectrometry [29,86].

## 6. Mechanisms of Action

Polyphenols possess various antimicrobial properties against bacteria, fungi, and viruses. They can disrupt quorum sensing, leading to interference with cell membranes, enzyme inhibition, the generation of reactive oxygen species (ROS), metal ion binding, host immune response modulation, and viral entry and replication prevention [12,87]. These actions enable polyphenols to eliminate various microbial species effectively. Enzyme inhibition can disrupt metabolic pathways, while interference with cell membranes can potentially induce cell death. Microbial elements may suffer oxidative damage from ROS, leading to cell death. Furthermore, polyphenols can interfere with quorum-sensing signals, influencing gene expression and biofilm development. Polyphenols can enhance defenses against microbial infections by modulating the host’s immune response. The specific mechanisms involved may vary depending on the targeted microorganism, the molecular structure of the secondary metabolites, and the surrounding conditions. Polyphenols’ ability to disrupt microbe cell membranes contributes to their antibacterial efficacy [88]. Their interactions with lipid bilayers, membrane permeability, and disruption of membrane proteins release ROS, which damages membrane integrity and function, ultimately leading to cell lysis. This process can cause microorganisms to lose their internal contents and disrupt their biological functions. Research has highlighted polyphenols as effective antifungal, antiviral, and antibacterial agents, making them a promising area of study in anti-infective research.

**Figure 4:**
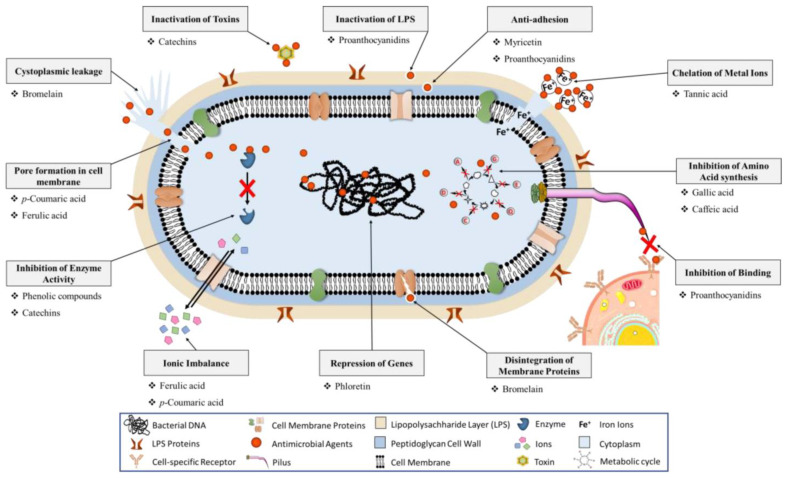
Antibacterial mechanism of polyphenols with their target sites [89].

Current research highlights the microbiological characteristics of various polyphenol extracts, both natural and synthetic. These extracts often show significant antibacterial effects against strains like streptococci, bacilli, and staphylococci. However, the exact mechanisms behind these effects remain not fully understood, complicated by the chemical diversity of polyphenols and the focus on whole extracts rather than isolated compounds. Studies suggest that the antibacterial effectiveness of polyphenols is linked to the structure of the molecules, particularly the quantity and positioning of the hydroxyl and methoxyl groups [87]. Gram-negative bacteria tend to be more resistant due to their less permeable lipophilic outer membrane and the potential degradation of the polyphenols by their enzymes. One proposed mechanism for this antimicrobial action involves the interaction of antioxidants with bacterial cell wall proteins, leading to membrane damage and cell death. Polyphenols can also inhibit biofilm production by disrupting quorum sensing and bacterial motility. Interestingly, they induce oxidative stress in bacteria, generating reactive oxygen species (ROS) that damage bacterial membranes. Additionally, polyphenols affect the biosynthesis of the critical proteins essential for bacterial metabolism, leading to irreversible metabolic changes and cell death. They inhibit key enzymes like DNA gyrase and ATP synthase, ultimately halting bacterial DNA synthesis and energy production [87]. Figure 4 presents the antibacterial mechanism of polyphenols with their target sites.

The mechanisms by which polyphenols act against fungi may play a role in blocking the efflux pump, affecting the cell membrane, interfering with ergosterol synthesis, damaging the cell wall, or generating reactive oxygen species (ROS). Numerous polyphenolic compounds have been examined for their antifungal properties. Recent research indicates that these compounds can influence the cell membrane and cell wall of fungi, potentially impacting ATP synthesis or the movement of Ca^++^ and K^+^ ions. Furthermore, polyphenols may inflict damage on the mitochondria of fungi, typically due to the effects of ROS. The disruption of the efflux pump has also been documented, and studies reveal that it impacts key proteins such as CDR1 or CDR2. Nonetheless, the most extensively investigated approach for developing antifungal agents has been targeting the fungal cell membrane [90].

**Figure 5:**
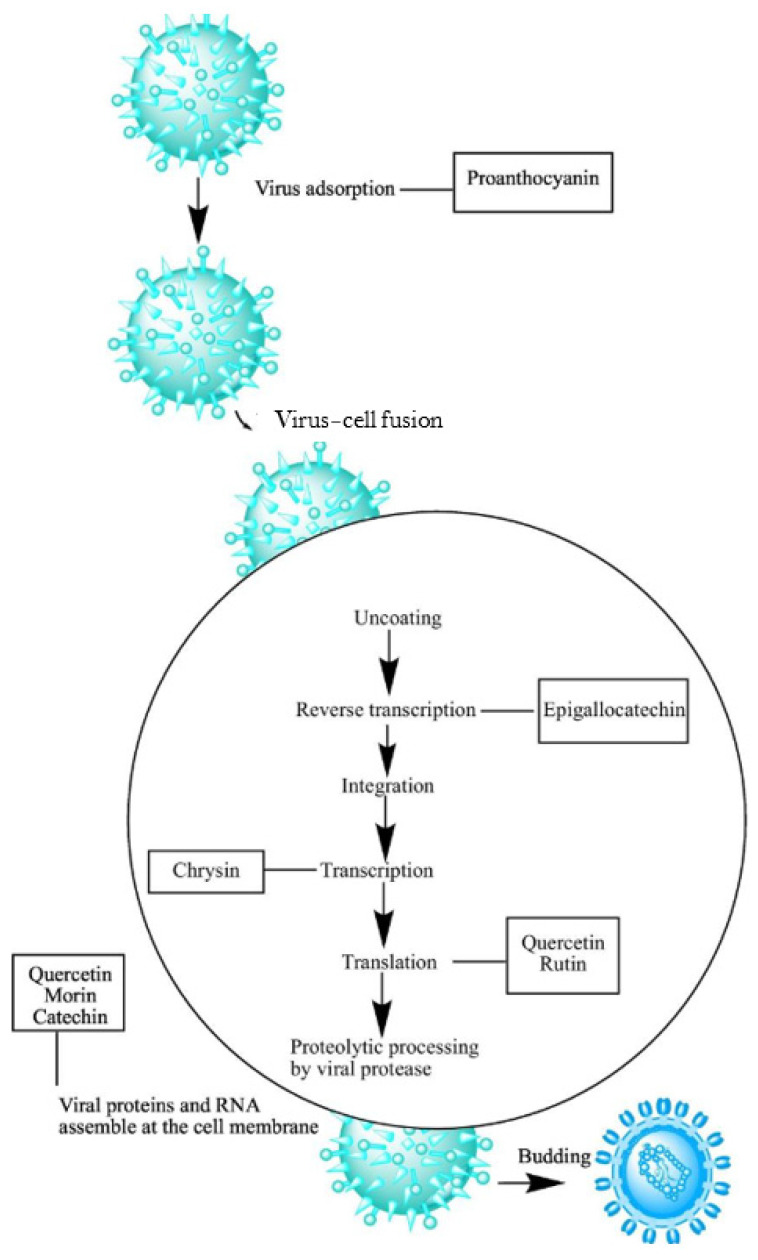
Scheme of the target sites of the antiviral mechanism of some polyphenols (e.g., quercetin, morin, chrysin) [77].

The mechanism by which polyphenols operate involves blocking the virus from entering the host cell. This was observed with the proanthocyanidins derived from *Rumex acetosa*, which hindered the entry of the influenza type A virus during its initial critical phase. The growing interest in polyphenols as antiviral agents is due to the significant benefits of utilizing nature-based compounds with minimal side effects on human health [77]. Polyphenols exhibiting antiviral properties may function through various mechanisms, such as obstructing viral entry and affecting replication. Emerging trends in biotechnology and medicine, along with novel processing technologies, could enhance solubility, administration, and therapeutic efficacy in preventing viral infections. Current research shows that polyphenols do not use just one specific mechanism to fight viruses. These bioactive compounds might exert their antiviral effects through various means, including antioxidant activities, blocking viral entry, hindering viral reproduction, and inhibiting DNA [77]. Figure 5 depicts some of the target sites of the antiviral mechanism of polyphenols like quercetin, morin, and chrysin.

## 7. Toxicity and Safety of Polyphenols

A study conducted by Boncler et al. [91] utilized the HCS assay to prioritize twenty-two polyphenol-rich compounds across five established cell lines, based on their in vitro toxicity profiles as determined by the total area under and above the dose–response curves measured for mitochondrial membrane potential, cell membrane permeability, and nuclear area. The analysis of cytotoxicity involved seventeen crude plant extracts and two commercial extracts (Aronox and Omnivir R), as well as three well-known phytochemicals that are commonly consumed by humans, including resveratrol, naringin, and kaempferol. Most of the compounds investigated (with naringin being the exception) showed varying toxicity profiles based on the methods used. Several polyphenol-rich compounds, such as spent hop extract, Aronox, and resveratrol, were classified as very safe or quite safe, like kale leaf and rowan fruit [91]. Their cytotoxic effects on mitochondrial membrane potential and plasma membrane integrity were found to be harmful when analyzing the nuclear area (notably resveratrol). Conversely, another group of extracts, including those from buckthorn bark, walnut husk, hollyhock flower, oak bark, oak leaf, silverweed herb, and blackberry leaf, exhibited high cytotoxicity in relation to mitochondrial membrane potential and cell membrane permeability, yet were relatively inert in causing nuclear morphology abnormalities. This phenomenon was particularly evident when the cumulative effects of the polyphenol-rich compounds were assessed. Moreover, kaempferol displayed significant cytotoxicity at both the mitochondrial and nuclear levels but only moderately impacted the plasma membrane permeability. The data presented underscores the necessity of carefully examining the toxicity of biologically active substances such as plant extracts [91].

There have been numerous reports of negative effects and toxicity in individuals after using green tea pills. The most prevalent side effects are gastrointestinal (nausea, diarrhea, bloating, and abdominal discomfort) and liver related [92]. Green tea polyphenols have shown negligible mutagenic or teratogenic effects in cell culture studies. Grape-derived polyphenols from seeds and skin have been tested in rats for 90 days and are safe at doses of up to 2.5% (*w*/*w*). Finally, anthocyanin-rich extracts were given orally to mice, rats, and guinea pigs at doses up to ten times the recommended level for human consumption for 90 days. They exhibited no toxic effects [92].

Matute et al. [93] investigated the antioxidant capabilities of 21 commercial fruit and vegetable juices using the ORAC assay (oxygen radical antioxidant capacity) and the “PMA-whole blood assay”, which produces the superoxide anion by stimulating whole blood with phorbol myristate acetate. In the PMA-whole blood assay, the juices containing pe-onidin-3-O-glucoside, epigallocatechin gallate, catechin, and quercetin inhibited superoxide anion generation at doses less than 1 µM [93].

**Figure 6:**
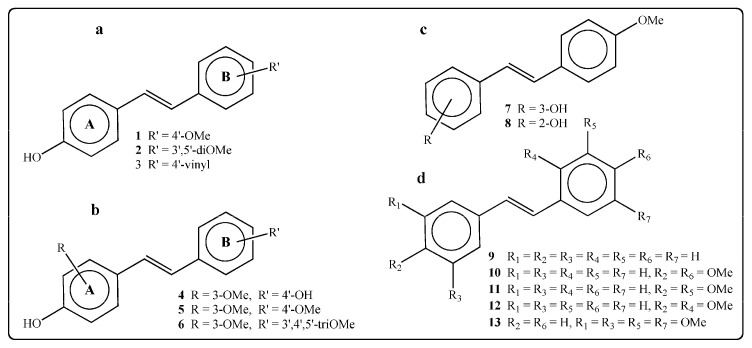
Structure of the stilbenes used for bioassays. (**a**) 4-OH stilbenes bearing substituents on cycle B; (**b**) 4-OH stilbenes bearing substituents on cycle A and/or cycle B; (**c**) structure of 2-OH and 3-OH stilbenes; (**d**) structure of stilbenes without phenolic function [94].

Chalal et al. [94] synthesized a series of 13 trans-resveratrol analogs in their study, via Wittig or Heck reactions, and assessed their antimicrobial activity on a grapevine pathogen responsible for severe diseases in the vineyard. Six of them, including the most active ones, were subsequently tested on the development of *Botrytis cinerea* (nectrotroph fungus responsible for gray mold) [94]. Figure 6 depicts the structure of stilbenes used for bioassays.

As reported by the authors, component **7** was the most effective against the pathogen demonstrating the inhibition of 50% of the mycelial growth (IC50) in 28 ± 3 µM [94].

Polyphenols can bind to transition metal ions (such as Fe and Cu), reducing the formation of free radicals in processes like Fenton and Haber–Weiss reactions. The strength of the bond between polyphenols and these ions differs depending on their molecular structure, the state of the ion, and the pH level [52]. This ability to chelate is advantageous as it decreases the production of free radicals and is utilized in treating iron overload, a factor in chronic diseases. Conversely, in cases of iron deficiency, polyphenols may hinder iron absorption in the gut, which can result in anemia and disrupt the balance of iron in the body [52].

Flavonoids can attach to proteins through nonspecific interactions and covalent links, creating complexes that affect the proteins’ functionality, structure, and stability. This interaction can influence how digestible and utilizable food proteins are, and it may modify the performance of digestive enzymes such as amylases, proteases, and lipases [52]. Although inhibiting certain enzymes may be advantageous in cases like diabetes or obesity, disruptions in enzyme function can result in undesirable digestive issues and impede nutrient absorption, negatively affecting overall health. The adverse effects of polyphenols on digestive system functionality may arise from the suppression of digestive enzymes and their effect on the gut microbiota. The suppression of intestinal microbiota by polyphenols leads to alterations in their physiological functions, such as the production of various bacterial metabolites crucial for human health, including vitamins, amino acids, short-chain fatty acids, antimicrobial peptides, and neurotransmitters. Additionally, the role of microbiota in detoxification is disrupted, as these microorganisms are responsible for breaking down food components and various xenobiotics (like drugs) [52].

Polyphenols interact with reactive oxygen species and various chemical compounds, negatively affecting human health. Notably, they can interact with drugs, such as iron supplements for anemia, influencing drug metabolism and pharmacokinetics, leading to altered therapeutic effects [52]. This occurs through their impact on drug-metabolizing enzymes, including cytochrome and others involved in phase I and II metabolism. Polyphenols can inhibit or reduce enzyme activity, affecting drug absorption, distribution, and metabolism. Increased drug concentration in the blood can be harmful, while increased metabolism can shorten drug efficacy. Additionally, polyphenols may influence drug transport by interacting with transporters like P-glycoprotein and organic ion transporters [52].

In certain instances, polyphenols may adversely affect cells, promoting mutagenesis or having cancer-causing and genotoxic consequences. Numerous studies indicate that some flavonoids can facilitate DNA cleavage through prooxidant activity and their interaction with topoisomerase IIα and Iiβ [52].

## 8. Future Perspectives

New polyphenolic systems with antimicrobial properties are still being researched in response to the increasing antibiotic resistance in traditional treatment. While concerns about stability may limit the effectiveness of polyphenols as alternatives to antibiotics for treating long-lasting wounds, they are still prospective. Efforts are being made to enhance efficiency by delivering bioactive polyphenols as natural antimicrobial agents to stabilize their use for complex wounds and improve wound healing. In the early stages of chronic wounds, the primary microorganisms identified are *S. aureus* and methicillin-resistant *S. aureus*, while *E. coli* and other pathogens are found later as the disease progresses. The polyphenolic compounds that support wound healing include kaempferol, chlorogenic acid, resveratrol, and ferulic acid. Tannic acid has several beneficial features that aid in wound treatment [95]. Recently, an antioxidant and antimicrobial hydrogel was developed to combat various pathogens in chronic wounds. A recent study has confirmed the wound-healing effects of tannic acid in both laboratory and animal tests, demonstrating its ability to stimulate hair follicles and accelerate skin regrowth. This suggests that tannic acid could be valuable in natural wound care materials. Results indicate that while pectin- and nanoparticulate-based systems improve the distribution of resveratrol by addressing its solubility and bioavailability issues, the microencapsulation of polyphenols enhances cutaneous wound healing with curcumin. Resveratrol in capsules exhibits more significant antimicrobial action than its natural form [12]. Polymeric nanoparticles based on polyphenols have recently become prominent in biomedical engineering, particularly as carriers for delivering drugs. Polyphenol nanoparticles can accommodate a range of drugs, including small molecule drugs, protein drugs, and nucleic acids, based on their structures and properties, to treat different diseases. According to a study conducted by Wang and colleagues, polyphenol nanoparticles, even without any payload, have shown promising therapeutic effects on the diseases associated with inflammation [96].

The solubility of polyphenols must be increased to increase the targeted and controlled release of bioactive polyphenols against microbial organisms. Modifying the delivery systems with a sustained release nature of drug molecules and polyphenols provides strong protection against microorganisms [97]. The naturally occurring polyphenols extracted from fruits, leaves, and algae show lower solubility in aqueous medium. Increasing the solubility of the polyphenols by converting them into salts can be more useful in different food and biomedical applications [98]. Creating a more soluble nature of these polyphenolic compounds through physiochemical modifications using positively charged metal ions can demonstrate superior effectiveness against harmful microorganisms and upgraded medicinal chemistry. It is essential to consider that the antibacterial, antifungal, antiviral, antioxidant, and anticancer activity of these polyphenols can vary based on various factors, including the polyphenol quantity, the solubility in different solvents, the type of bacteria, fungi, and viruses being targeted, and the tested delivery method. Multiple approaches may be involved in how polyphenolic systems exhibit their antimicrobial properties compared to pure and modified bioactive polyphenolic substances, which are still under investigation [12].

Polyphenols are generally safe for healthy individuals when consumed in a diverse and balanced diet. These natural compounds are beneficial for preventing and treating various diseases and combating different types of stress. Therefore, it is recommended to incorporate polyphenols into our diets, mainly by consuming natural, minimally processed foods such as fruits, vegetables, nuts, herbs, spices, tea, and juices. However, potential adverse effects increase when polyphenols are consumed in large quantities without medical supervision, such as dietary supplements or plant extracts. These products often contain high doses of polyphenols, typically chemically purified aglycones (as opposed to glycosides found in natural foods), which cannot be obtained through normal dietary intake. The adverse effects of polyphenols are also more likely to occur when individuals with chronic illnesses who are taking prescribed medications consume a polyphenol-rich diet or supplements [52].

Nanotechnology, especially nano-based carriers, has emerged as a viable solution to address the challenges of polyphenols’ limited bioavailability. The small dimensions of nanoparticles promote improved absorption and the prolonged release of polyphenols. Research is concentrating on lipid-based materials, dendrimers, and polymeric nanoparticles due to their biodegradable nature and modifiable properties [99]. A variety of nanocarriers, such as micelles, cyclodextrins, and gelatin, serve to protect polyphenols. Both organic nanoparticles (derived from proteins and carbohydrates) and inorganic ones (such as gold, silver, and silica) have been utilized to boost the stability and bioavailability of hydrophobic polyphenols like curcumin and catechin through methods like nanoencapsulation. Techniques involving biopolymer-based nanoencapsulation and using natural nanocarriers are currently being investigated to overcome absorption obstacles and enhance the delivery of these compounds [99].

## 9. Conclusions

Natural plant polyphenols and their synthetic analogs are considered as some of the potential routes to attempt tackling the pressing problem of antibiotic resistance. Being powerful against many clinically significant Gr (+) and Gr (−) pathogenic bacteria, fungi like *C. albicans* and *C. neformans* cause serious infections at low MIC, making them potential routes for biotechnological applications in medicine molecules. Their broad antiviral activity towards HCV, HIV, FLU, DENV and SARS coronaviruses pave the path for future innovative applications of polyphenols. Targeting plenty of the vital processes in bacteria, fungi, and viruses, these molecules successfully disrupt cells and virus particles. However, their positives are overshadowed by their negative cytotoxic effects on mitochondrial and plasma membranes, hindering iron absorption in the gut, which often results in anemia. At the molecular level, researchers have focused on the promoters of mutagenesis and genotoxicity, cancer causers, the facilitators of DNA cleavage, and the retardants of drug transport in an attempt to diminish them. Biotechnological advances, especially the entrapment of polyphenols in bio-compatible nanoparticles to enhance their bioavailability and efficacy and fast delivery have become the targets of vast interest. Polyphenols are promising for exploring molecules as novel antimicrobial substances paving the path for effective novel antimicrobial agents’ discovery, taking into consideration both their positives and negatives.

## Figures and Tables

**Table 1:** Foods of daily diet supplying polyphenols [29], adapted after [30,31].

Vegetables	Fruits	Grains	Beans	Herbs and Spices	Beverages
Artichoke	Apples	Oat	Black beans	Basil	Black tea
Asparagus	Apricots	Rye	Soy meat	Black tea	Coffee
Broccoli	Black chokeberry	Whole grains	Soy milk	Celery	Dark chocolate
Capers	Black currant	Wheat	Sprout	Cinnamon	Ginger
Carrots	Black elderberry		White beans	Cumin	Green tea
Cayenne pepper	Black grapes			Curry	Olive oil
Garlic	Blackberry			Ginger	Rapeseed oil
Olives	Blueberry			Green tea	Red wine
Potatoes	Cherry sour			Marjoram	Vinegar
Red lettuce	Cherry sweet			Oregano	
Onion	Grapefruit			Parsley	
Spinach	Nectarines			Peppermint	
	Peaches			Rosemary	
	Pears			Sage	
	Pomegranate			Spearmint	
	Plum			Star anise	
	Raspberry				
	Strawberry				

**Table 2:** Antibacterial activity of polyphenol-containing vegetables (inhibition zones d in mm) against *B. subtilis* and *E. coli* type strains, personal results [29].

Vegetable/Plant Vegetative Organ	Inhibition Zones on *B. subtilis* NIBMCC 8752	Inhibition Zones on *E. coli* NIBMCC 8751
Parsley (leaves)	2	0
Tomato (seeds)	5	0
Cayenne pepper (tissue disks)	24	25
Cayenne pepper (seeds)	7	11
Onion yellow skin (mature bulbs)	27	3
Onion red skin (mature bulbs)	25	3
Onion young (fresh bulbs)	0	0
Garlic (mature bulbs)	7	30
Garlic young (fresh bulbs)	2	0

## Data Availability

The original contributions presented in the study are included in the article, further inquiries can be directed to the corresponding author.
